# Opposite Profiles of Complement in Antiphospholipid Syndrome (APS) and Systemic Lupus Erythematosus (SLE) Among Patients With Antiphospholipid Antibodies (aPL)

**DOI:** 10.3389/fimmu.2019.00885

**Published:** 2019-05-07

**Authors:** Stephanie L. Savelli, Robert A. S. Roubey, Kathryn J. Kitzmiller, Danlei Zhou, Haikady N. Nagaraja, Evan Mulvihill, Fatima Barbar-Smiley, Stacy P. Ardoin, Yee Ling Wu, Chack-Yung Yu

**Affiliations:** ^1^The Research Institute at Nationwide Children's Hospital, Columbus, OH, United States; ^2^Division of Hematology/Oncology, Nationwide Children's Hospital, Columbus, OH, United States; ^3^Department of Pediatrics, College of Medicine, The Ohio State University, Columbus, OH, United States; ^4^Division of Rheumatology, Allergy and Immunology, The University of North Carolina at Chapel Hill, Chapel Hill, NC, United States; ^5^Division of Rheumatology, Nationwide Children's Hospital, Columbus, OH, United States; ^6^Division of Biostatistics, College of Public Health, The Ohio State University, Columbus, OH, United States; ^7^Department of Microbiology and Immunology, Loyola University Chicago, Maywood, IL, United States

**Keywords:** C3 and C4, C4A and C4B, Copy number variation, Factor H, Lupus anticoagulant, Mannan binding lectin, Recurrent pregnancy loss, Thrombosis

## Abstract

APS is the association of antiphospholipid antibodies (aPL) with thromboses and/or recurrent pregnancy loss (RPL). Among patients with SLE, one-third have aPL and 10–15% have a manifestation of secondary APS. Animal studies suggested that complement activation plays an important role in the pathogenesis of thrombosis and pregnancy loss in APS. We performed a cross-sectional study on complement proteins and genes in 525 patients with aPL. Among them, 237 experienced thromboses and 293 had SLE; 111 had both SLE and thromboses, and 106 had neither SLE nor thrombosis. Complement protein levels were determined by radial immunodiffusion for C4, C3 and factor H; and by functional ELISA for mannan binding lectin (MBL). Total *C4, C4A* and *C4B* gene copy numbers (GCN) were measured by TaqMan-based realtime PCR. Two to six copies of *C4* genes are frequently present in a diploid genome, and each copy may code for an acidic C4A or a basic C4B protein. We observed significantly (a) *higher* protein levels of total C4, C4A, C4B, C3, and anticardiolipin (ACLA) IgG, (b) increased frequencies of lupus anticoagulant and males, and (c) decreased levels of complement factor H, MBL and ACLA-IgM among patients with thrombosis than those without thrombosis (*N* = 288). We also observed significantly *lower* GCNs of total *C4* and *C4A* among aPL-positive patients with both SLE and thrombosis than others. By contrast, aPL-positive subjects with SLE had significantly reduced protein levels of C3, total C4, C4A, C4B and ACLA-IgG, and higher frequency of females than those without SLE. Patients with thrombosis but *without* SLE (*N* = 126), and patients with SLE but *without* thrombosis (*N* = 182) had the greatest differences in mean protein levels of C3 (*p* = 2.6 × 10^−6^), C4 (*p* = 2.2 × 10^−9^) and ACLA-IgG (*p* = 1.2 × 10^−5^). RPL occurred in 23.7% of female patients and thrombotic SLE patients had the highest frequency of RPL (41.0%; *p* = 3.8 × 10^−10^). Compared with non-RPL females, RPL had significantly higher frequency of thrombosis and elevated C4 protein levels. Female patients with homozygous C4A deficiency *all* experienced RPL (*p* = 0.0001) but the opposite was true for patients with homozygous C4B deficiency (*p* = 0.017). These results provide new insights and biomarkers for diagnosis and management of APS and SLE.

## Introduction

Antiphospholipid syndrome (APS) is characterized by vascular thrombosis and/or pregnancy morbidity such as recurrent fetal loss in the persistent presence of antiphospholipid antibodies (aPL) (1–7). aPL are a heterogeneous group of autoantibodies that include antibodies against phospholipid binding protein β_2_-glycoprotein I (β_2_GPI), anticardiolipin antibodies (ACLA), and lupus anticoagulant (LAC) (8). Human subjects with triple positivity for all three groups of aPL appeared to be at high risk to experience recurrent thromboembolic events (9). A majority of clinical tests for aPL detects antibodies against β_2_GPI. β_2_GPI is a plasma protein consisting of five structural domains known as short consensus repeats that are characteristic features of controlling proteins for the complement system (10–12).

SLE is a common autoimmune disease associated with APS. SLE features the generation of autoantibodies against nuclear antigens including double-stranded DNA (13, 14). In a study of European APS patients, over 40% were found to have SLE or a lupus-like disease (15). Among the general SLE population, between 30 and 40% have aPL; and 10–15% of patients with SLE also have clinical manifestations of APS (15–20). In addition to the presence of autoantibodies, *hypo*complementemia is another hallmark of human SLE (21–25). Low serum complement levels for C4 and C3 in patients with SLE can be triggered by a combination of heritable and acquired factors: genetic deficiencies, low copy number of complement *C4* genes, robust consumption caused by immune complex-mediated complement activation, or the presence of inhibitors that inactivate or prevent accessibility. A complete genetic deficiency in any one of the early components specific for the classical complement activation pathway *almost always* lead to pathogenesis of human SLE, inferring that an intact classical pathway of the complement system is essential for the protection against systemic autoimmunity (26–28).

Activations of complement C3 and C5 in the presence of antigen-antibody complexes occur via the formation of the C1 complex (C1q-C1r_2_-C1s_2_), followed by the activations of C4 and C2 to form C4b and C2a, respectively (29). C4b and C2a are subunits of the C3 and C5 convertases, essential for the classical and lectin activation pathways (26). There are two isotypes of native C4 proteins. C4A is the acidic isotype believed to play an essential role in immune clearance and immunotolerance. C4B is the basic isotype that is capable of rapid propagation of complement activation (30–34). In a diploid genome, complement *C4* gene copy number varies among different individuals. Two to eight copies of *C4* genes are generally present in a diploid genome among most human subjects (35, 36). Each *C4* gene either codes for a C4A or a C4B protein. Such gene copy number variation contributes to quantitative and qualitative diversities in C4 protein levels and function, and therefore different intrinsic strengths for effector functions of innate and adaptive immune responses (25, 34, 36–40). Among European and East-Asian subjects, low copy number of total *C4* or *C4A* is a risk factor for SLE, while high copy number of total *C4* or *C4A* is protective against susceptibility to SLE (22, 38, 41, 42).

An injection of human aPL into animal models including wild-type *mice* induced an increase in thrombus size (43, 44). An injection of human aPL into pregnant mice resulted in fetal resorption. (45, 46). Mice *deficient* in complement C3 or C5, as well as mice injected with a monoclonal antibody against C5, did *not* exhibit an increase in thrombus size in the presence of aPL. Blockade of complement activation by genetic deletion of C3 or C4, or with transgenic insertion of complement regulatory protein Crry-Ig, a soluble inhibitor of mouse C3 convertase, *protected* mice, rats or hamsters from pregnancy complications induced by injections of human aPL (45, 47–56). These phenomena suggest that complement proteins or their activated products are engaged in the pathogenesis of APS, as they probably provide immune effectors for aPL-mediated thromboses, tissue injury and/or fetal loss in mouse models. The generation of immune complexes between aPL and ligands (such as β_2_GPI binding to phospholipids) leads to activation of the complement classical pathway, release of C5a and C3a anaphylatoxins (50, 57, 58), which may attract neutrophils and other granulocytes to the site of complement activation, increase vascular permeability, and elicit inflammatory response that contributes to tissue injuries including pregnancy morbidity. Culmination of complement activation pathways leads to the assembly of the membrane attack complex (C5b-9) and provides the “second-hit” to trigger vascular thrombosis (53). Consistent with this notion, it was shown that C3, C5, or C6-deficient rodents were protected from aPL induced thrombosis (56, 59). Such protective effects of complement deficiency in APS-associated disorders observed in animal models are opposite to the causal effects of deficiencies in early components for the classical complement pathway in human lupus (26, 60). Among human patients with APS, elevated levels of complement activation products (C4a, C3a, C5a, C5b-9) have been demonstrated (55, 61, 62). However, systematic and meticulous studies on how complement proteins and genes contribute to the pathology of human APS (recurrent vascular thrombosis or pregnancy morbidity) and the concurrence of SLE and APS were scarce or limited by small sample size.

Here we performed a cross-sectional study on 525 human subjects with aPL from the Antiphospholipid Syndrome Collaborative Registry. Based on clinical presentations of thrombosis and SLE, these subjects were categorized to four groups: patients with thrombosis only (T_o_), with thrombosis and SLE (TS), with SLE only (S_o_), and without thrombosis and without SLE (NTS). Plasma protein concentrations for complement total C4, C4A, C4B, C3, factor H, and functional mannan binding lectin (MBL) were measured. Total *C4, C4A* and *C4B* gene copy numbers were elucidated. The results reveal substantial phenotypic differences for complement protein concentrations among patients with thromboses or recurrent pregnancy loss, and SLE. There was also a significant difference in *C4* gene copy number variations between patients with both thrombosis and SLE, and patients without SLE and thrombosis.

## Patients and Methods

### Study Population

This study was approved by the Institutional Review Board at Nationwide Children's Hospital. Peripheral blood plasma and matched genomic DNA samples without personal identifiers from 525 patients with aPL and clinical status were provided by the APS Core at University of North Carolina (8, 63). These aPL-positive patients were recruited with written informed consent. Of these patients, 444 (84.57%) were female and 81 (15.43%) were male. The mean age (±SD) was 45.01 ± 12.97 years old. Among these aPL-positive patients, 184 (35.05%) met the Sapporo criteria for definite APS (1); an additional 175 subjects (33.33%) met the extended definition of APS, and 166 asymptomatic subjects (31.6%) who had aPL but no manifestations of thrombosis or pregnancy morbidity. Patients with definite APS as defined by the preliminary or modified Sapporo criteria (1, 6) must have one or more clinical episodes of vascular thrombosis and/or pregnancy morbidity as well as ACLA, anti-β_2_GPI IgG and/or IgM or LAC present on two or more occasions at least 6 weeks apart. The expanded APS group was defined by institutions participating in APSCORE and include those patients with one or more clinical manifestations characteristic of APS but not fulfilling the strict definition and either the Sapporo laboratory criteria or one of a group of APS-associated autoantibodies. Asymptomatic patients fulfill the Sapporo laboratory criteria but have no clinical manifestations related to APS.

To perform refined analyses based on clinical presentations, we segregated the aPL subjects based on the presence and absence of thrombosis, SLE and recurrent pregnancy loss. Among the study cohort, 237 subjects had a history of thrombosis and 288 subjects did not have thrombosis. A total of 293 subjects were diagnosed with SLE according to the American College of Rheumatology criteria (64) and 232 subjects did not have a diagnosis of SLE at the time of recruitment. Of the 444 female subjects with aPL, 106 (23.87%) experienced recurrent pregnancy loss.

### Quantifications of Total *C4, C4A*, and *C4B* Genes by Real-Time PCR

A series of real-time PCR assays was applied to determine the copy number variations of total *C4, C4A*, and *C4B* genes (35). All real time PCR assays used the TaqMan MGB probes (ABI). The target probes (*C4, C4A*, and *C4B*) were VIC-labeled. The endogenous control probe, which targeted an invariant exon 4 of the *RP1* gene, was FAM-labeled. Each reaction consisted of 0.5 to 1 μM of both forward and reverse primers for the target and control amplicons, 100 nM of the target and endogenous control probes, 25 ng of sample DNA and TaqMan Universal PCR master mix (ABI, PN 4323018). All assays were performed in triplicates using the ABI 7500 RT-PCR system per manufacturer's recommendations. The relative standard curve method was utilized to calculate the copy number of each target gene. The accuracy of *C4A* and *C4B* gene copy number assignments for each sample was cross-confirmed as the gene copy number of total *C4* equals the sum of *C4A* and *C4B*.

### Complement C3, C4, Factor H (CFH) and Mannan Binding Lectin (MBL) Protein Concentrations in Citrate-Plasma

Platelet poor plasma samples were processed with a consistent protocol. Briefly, blood samples in citrate tubes were centrifuged at 1,500 *g* for 10 min, at 4–8°C. Plasma samples were transferred to microcentrifuge tubes and spun again at 2,000 *g* for 5 min. Aliquots were kept frozen at −80°C. Plasma protein concentrations of complement C3 and C4 were determined by single radial immunodiffusion assays using commercial kits from The Binding Site (Birmingham, United Kingdom). A comparison of C4 protein concentrations of (a) SLE patients without thrombosis from this study and (b) an *independent* cross-sectional study of Ohio SLE (38) revealed that protein concentrations of complement C4 assayed from platelet-poor citrate plasma, which were subjected to two rounds of centrifugation, were ~14.5% *lower* than that of EDTA-plasma C4 harvested after a single round of centrifugation.

Complement factor H plasma protein concentrations were measured using homemade RID plates according to a standard protocol (65). Plasma concentrations for MBL were determined using a functional assay kit from the Antibody Shop (Denmark).

### Complement C4 Protein Allotyping

Plasma C4A and C4B protein allotypes were determined by immunofixation and immunoblot techniques, as described previously (66–68). The relative band intensities of C4A and C4B allotypes from each sample were quantified by ImageQuant Software. The corresponding plasma C4A and C4B protein concentrations were calculated from the total C4 protein concentrations.

### Statistical Analyses

Descriptive statistics, including means, standard deviations (SD), and 95% confidence intervals (95% CI) were computed for numeric data, and frequency distributions were determined for categorical variables, using statistical software JMP13 (SAS Institute) and GraphPad Prism6 software. Two group comparisons were based on *t*-tests that accounted for unequal variances if appropriate. Specifically, Tukey HSD test with an alpha set at 0.05 was applied, and was followed by pairwise Student's *t*-tests that yielded *p*-values. Dunnett's test with an alpha of 0.05 was applied for comparing study groups to controls. Categorical data were compared by χ^2^ analyses and odds ratios were calculated whenever appropriate.

To allow a standardized comparison of all continuous parameters contributing to thrombosis without SLE, SLE without thrombosis, SLE with thrombosis, and no thrombosis and no SLE, we determined the root mean square error (RMSE) of each parameter in these four groups by analysis of variance (ANOVA). The difference in the mean protein levels for each protein between any two groups divided by its RMSE to give the effect size index (69). The mean values of parameters in the NTS group were used as references and the *effect size indices* for T_o_, S_o_, and TS groups were graphically plotted. This enabled a uniform depiction on effects of all continuous parameters under different clinical conditions.

Stepwise multiple logistic regression analyses were used to identify independent parameters significant for clinical outcomes: thrombosis, arterial thrombosis, venous thrombosis, pregnancy loss, and SLE. Such analyses allowed controlled studies for continuous and categorical parameters. For analyses of a clinical presentation as a response, we first put all parameters studied [i.e., C3 or C4, factor H, MBL, ACLA-IgM, ACLA-IgG, BMI, age, gender (F/M), LAC (presence or absence), SLE (presence or absence)] into the regression model. Those that did not give a *p*-value smaller than 0.1 were removed from the subsequent analyses. The last best model with parameters represented by *p*-values smaller than 0.05 was maintained and presented. Unit Odds Ratio (OR) and range OR were computed. Parameters that could not coexist in the regression model because of strong correlation (e.g., C3 and C4) were put into the regression models separately and the stronger parameter was kept. When C4 was identified as a significant parameter in a model, we further asked whether C4A or C4B or both C4 isotypes were playing a major role.

## Results

The study population consisted of 525 human subjects with antiphospholipid antibodies (aPL), recruited through the APSCORE. The mean age of subjects at the time of recruitment was 45.0 ± 13.0 (mean ± SD) years old. These study subjects were initially segregated into three groups: definite APS, extended APS, and non-APS based on clinical manifestations associated with APS, which included vascular thromboses and pregnancy morbidity. Results for an initial characterization for these three groups of patients are shown in Table 1. One remarkable feature emerged was the steady and highly significant increase in the mean protein concentration of complement C4 from non-APS (16.6 ± 8.8 mg/dL), to extended APS (20.0 ± 9.2 mg/dL), and definitive APS (22.9 ± 10.0 mg/dL) (*p* = 2.6 × 10^−8^). These three groups of patients had different frequencies of SLE, thromboses and pregnancy morbidities. Thus, we set to examine quantitative variations of plasma complement proteins among aPL subjects with different clinical manifestations for thrombosis and/or SLE in both female and male patients, and recurrent pregnancy loss among female patients. The demographic and clinical features for these aPL-positive subjects are shown in Table 2.

**Table 1:** Demographics of study populations: aPL patients with definite APS, extended APS and non-APS.

	**Definite APS**	**Extended APS**	**Non-APS**	p^*^
n (%)	184 (35.1)	175 (33.3)	166 (31.6)	
Sex, F/M (ratio)	147/37 (4.20:1)	152/23 (6.61:1)	145/21 (6.90:1)	0.099
Age ± SD; years old	44.40 ± 12.54	46.82 ± 13.09	43.78 ± 13.19	0.071
Race: White/Black/Others,^||^*n* (% in each group)	81/24/79 (44.0/13.0/42.9)	67/27/81 (38.3/15.4/46.3)	80/23/63 (48.2/13.9/38.0)	0.44
BMI	28.66 ± 6.86	28.94 ± 7.58	28.22 ± 6.57	0.64
Thrombosis, Y/N, *n* (%)	154/30 (83.7)	83/92 (47.4)	0/166 (0)	**1.4** × **10**^**−69**^
Pregnancy loss, female, Y/N, *n* (%)	80/67 (54.4)	26/126 (17.1)	0/166 (0)	**1.7** × **10** ^**−32**^
SLE, Y/N, *n* (%)	89/95 (48.4)	99/76 (56.6)	105/61 (63.3)	**0.019**
Complement C3 ± SD; mg/dL	124.1 ± 36.0	128.4 ± 37.8	118.8 ± 33.4	**0.048**
Complement C4 ± SD; mg/dL	22.9 ± 10.9	20.0 ± 9.2	16.6 ± 8.8	**2.6** × **10**^**−8**^
Correlations between C3 and C4, *r*^2^	0.217 (*p* = **3.0** **×** **10**^**−11**^)	0.310 (*p* = **2.7** **×** **10**^**−15**^)	0.321 (*p* = **4.0** **×** **10**^**−15**^)	

*r, coefficient of correlation*.

* *p values obtained by χ^2^ analyses for categorical data, or by ANOVA (analysis of variance) for continuous data; ^||^others: other racial and multi-racial groups; p- < 0.05 are in bold fonts*.

**Table 2:** Demographic data and disease status of aPL-positive subjects.

***A. Thrombosis status***					
	**T**	**NT**	***p***		
*N*	237	288			
Age	46.4 ± 13.4	43.9 ± 12.5	**0.029**		
Sex			**7.2 × 10**^**−6**^		
F	182 (0.768)	262 (0.910)			
M	55 (0.232)	26 (0.090)			
F/M ratio	3.21	10.1			
BMI	29.6 ± 7.6	27.8 ± 6.4	**0.0033**		
Race			0.72		
White	107 (0.452)	121 (0.420)			
Black	31 (0.131)	43 (0.149)			
Others	99 (0.418)	124 (0.431)			
***B. SLE status***					
	**S**	**NS**			
N	293	232			
Age	44.6 ± 12.4	45.5 ± 13.7	**0.42**		
Sex			0.0031		
F	260 (0.884)	184 (0.793)			
M	33 (0.113)	48 (0.207)			
F/M ratio	7.88	3.83			
BMI	28.7 ± 7.3	28.5 ± 6.7	0.76		
Race, *n* (frequency in each group)				**7.0 × 10**^**−7**^	
White	107 (0.365)	121 (0.522)			
Black	60 (0.205)	14 (0.060)			
Others	126 (0.430)	97 (0.418)			
***C. Thrombosis and SLE status***					
	**T**_**o**_	**TS**	**S**_**o**_	**NTS**	**p**
*N*	126	111	182	106	
Age	47.7 ± 13.9	44.9 ± 12.8	44.4 ± 12.1	43.0 ± 13.1	**0.041**
Sex					**1.2 × 10**^**−7**^
F	85 (0.675)	97 (0.874)	163 (0.896)	99 (0.934)	
M	41 (0.325)	14 (0.126)	19 (0.104)	7 (0.066)	
F/M ratio	2.07	6.93	8.58	14.1	
BMI	29.7 ± 7.2	29.5 ± 8.0	28.2 ± 6.7	27.0 ± 5.8	**0.014**
Race					**2.9 × 10**^**−6**^
White	60 (0.476)	47 (0.423)	60 (0.330)	61 (0.576)	
Black	11 (0.087)	20 (0.180)	40 (0.220)	3 (0.028)	
Others	55 (0.436)	44 (0.396)	82 (0.451)	42 (0.396)	
Pregnancy loss					**2.0 × 10**^**−10**^
yes	24 (0.282)	44 (0.448)	14 (0.086)	24 (0.242)	
no	61 (0.718)	53 (0.552)	149 (0.914)	75 (0.758)	
**Correlations between C3 and C4**					
r^2^	0.167	0.212	0.320	0.352	
p	**2.0 × 10**^**−6**^	**5.3 × 10**^**−7**^	**2.5 × 10**^**−16**^	**2.5 × 10**^**−11**^	

*Key: BMI, body mass index; NS, no SLE; NT, no thrombosis; NTS, no thrombosis and no SLE; S, SLE; S_o_, SLE without thrombosis; T, thrombosis; T_o_, thrombosis without SLE; TS, thrombosis and SLE; frequency in each group is shown in parenthesis. p-values of statistical significance are in bold fonts*.

### Variations of Plasma Complement Protein and ACLA Levels in aPL-Positive Subjects With and Without Thromboses

Among the aPL-positive subjects, 45.1% had a past history of thrombosis, and 55.8% were diagnosed with SLE at the time of recruitment. When the mean plasma complement protein concentrations and aPL between thrombotic and non-thrombotic groups were compared, highly significant phenotype differences for total C4, C4A, C4B, MBL, ACLA-IgM, and ACLA-IgG were observed (Table 3). The mean protein level (and 95% confidence interval) for total C4 was 22.7 (21.3–24.0) mg/dL in the thrombotic group, and 17.7 (16.6–18.7) mg/dL in the non-thrombotic group, which represented a difference of 28.2% (*p* = 1.3 × 10^−8^).

**Table 3:** Mean plasma complement and ACLA protein levels in aPL-positive subjects with (T) and without thrombosis (NT).

	***n***	**Mean ± SD**	**95% CI**	***p* (NT vs. T)**
C3 protein (mg/dL)				**0.013**
NT	284	120.2 ± 35.1	116.1–124.3	
T	236	128.1 ± 36.6	123.4–132.8	
C4 protein (mg/dL)				**1.3** **×** **10**^**−8**^
NT	282	17.7 ± 9.0	16.6–18.7	
T	235	22.7 ± 10.6	21.3–24.0	
C4A protein (mg/dL)				**8.2** **×** **10**^**−6**^
NT	273	9.6 ± 5.6	8.9–10.3	
T	233	12.1 ± 6.7	11.2–12.9	
C4B protein (mg/dL)				**6.7** **×** **10**^**−6**^
NT	283	8.3 ± 5.0	7.8–8.9	
T	234	10.5 ± 6.0	9.8–11.3	
CFH protein (mg/dL)				0.057
NT	249	52.3 ± 15.0	50.5–54.2	
T	207	49.9 ± 12.1	48.2–51.5	
MBL protein				**0.0007**
NT	242	0.167 ± 0.173	0.145–0.189	
T	214	0.117 ± 0.129	0.100–0.135	
ACLA IgM protein (MPL)				**0.0022**
NT	254	29.9 ± 37.5	25.3–34.6	
T	202	19.5 ± 33.2	14.9–24.1	
ACLA IgG protein (GPL)				**0.0043**
NT	254	29.6 ± 46.7	23.9–35.4	
T	206	45.1 ± 68.4	35.7–54.5	
			**Odds ratio (95% CI)**	***p***
LAC-Positivity (freq.)			2.63 (1.76–3.94)	**1.3** **×** **10**^**−6**^
NT	260	0.531		
T	199	0.749		
Sex (F/M ratio)			0.33 (0.20–0.54)	**7.2** **×** **10**^**−6**^
NT	288 (262/26)	10.0		
T	237 (182/55)	3.23		

*CI, confidence interval. Odds ratios were calculated for T vs. NT. p-values of statistical significance are in bold fonts*.

Parallel increases in complement C4A and C4B were observed in the thrombotic group. Mean concentration of plasma C4A in the thrombotic group was 12.1 (11.2–12.9) mg/dL, and 9.6 (8.9–10.3) mg/dL in the non-thrombotic group (*p* = 8.2 × 10^−6^). Plasma C4B mean protein concentration in the thrombotic group was 10.5 (9.8–11.3) mg/dL, and 8.3 (7.8–8.9) mg/dL in the non-thrombotic group (*p* = 6.7 × 10^−6^). For complement C3, moderately higher mean protein level was observed in the thrombotic group than the non-thrombotic group (T: 128.1 mg/dL; NT: 120.2 mg/dL; *p* = 0.013).

By contrast, mean plasma protein level for functional MBL among thrombotic subjects was significantly *lower* than that of non-thrombotic subjects, which were 0.117 (0.100–0.135) mg/dL and 0.167 (0.145–0.189) mg/dL, respectively (*p* = 0.0007). Slightly lower levels of factor H protein were also observed in the thrombotic group (T: 49.9 mg/dL; NT: 52.3 mg/dL; *p* = 0.057).

The mean values of anticardiolipn antibodies among thrombotic subjects were 19.5 (14.9–24.1) units for ACLA-IgM and 45.1 (35.7–54.5) units for ACLA-IgG. In non-thrombotic subjects, the corresponding values were 29.9 (25.3–34.6) and 29.6 (23.9–35.4) units (*p* = 0.0022 for ACLA-IgM; *p* = 0.0043 for ACLA-IgG). Thrombotic subjects had lower levels of ACLA-IgM but higher levels of ACLA-IgG. Three-quarters (74.9%) of thrombotic subjects tested positive for the presence of lupus anticoagulant (LAC), compared to slightly over one-half (53.3%) among non-thrombotic subjects (*p* = 1.7 × 10^−6^).

Thrombotic subjects had significantly lower female to male ratio (3.23 to 1) when compared with non-thrombotic subjects (10.0 to 1; *p* = 7.2 × 10^−6^).

### Plasma Complement Protein and ACLA Levels in aPL-Positive Subjects With and Without SLE

Quantitative variations of complement and ACLA plasma protein were compared between the aPL-positive patients with and without SLE (Table 4). The mean C3 concentrations were 118.3 (114.2–122.4) mg/dL in SLE and 130.8 (126.2–135.4) mg/dL in non-SLE, which represented a *reduction* of 9.6% of mean C3 level in SLE (*p* = 8 × 10^−5^). The mean total C4 concentrations were 18.6 (17.4–19.8) mg/dL in SLE and 21.6 (20.4–22.9) mg/dL in non-SLE, which corresponded to a reduction of 13.9% of total C4 in SLE (*p* = 0.0006). The mean C4A and C4B concentrations were 10.0 (9.2–10.8) mg/dL and 8.7 (8.1–9.4) mg/dL, respectively, in the SLE group; and were 11.6 (10.9–12.4) mg/dL and 10.1 (9.4–10.8) mg/dL, respectively, in the non-SLE group (*p* = 0.0038 for C4A; *p* = 0.005 for C4B). Thus, *lower* plasma levels of complement C3 and C4 were conspicuous in the SLE group.

**Table 4:** Mean plasma protein concentrations of complement and ACLA in aPL-positive subjects with (S) and without (NS) SLE.

	***n***	**Mean ± SD**	**95% CI**	***p* (NS vs. S)**
C3 protein (mg/dL)				**8.0** **×** **10**^**−5**^
NS	230	130.8 ± 35.4	126.2–135.4	
S	290	118.3 ± 35.6	114.2–122.4	
C4 protein (mg/dL)				**0.0006**
NS	230	21.6 ± 9.6	20.4–22.9	
S	287	18.6 ± 10.3	17.4–19.8	
C4A protein (mg/dL)				**0.0038**
NS	228	11.6 ± 5.8	10.9–12.4	
S	278	10.0 ± 6.5	9.2–10.8	
C4B protein (mg/dL)				**0.005**
NS	230	10.1 ± 5.4	9.4–10.8	
S	287	8.7 ± 5.7	8.1–9.4	
CFH protein (mg/dL)				**0.02**
NS	203	52.9 ± 14.4	50.9–54.9	
S	253	49.9 ± 13.1	48.3–51.5	
MBL protein				0.29
NS	204	0.135 ± 0.140	0.116–0.154	
S	252	0.151 ± 0.168	0.130–0.172	
ACLA IgM protein (MPL)				0.22
NS	183	22.8 ± 29.6	18.5–27.1	
S	273	27.0 ± 39.7	22.3–31.8	
ACLA IgG protein (GPL)				**0.0002**
NS	187	48.8± 70.1	38.7–59.0	
S	273	28.2 ± 46.1	22.7–33.7	
			**Odds ratio (95% CI)**	**p**
LAC-positivity (freq)			0.79 (0.54–1.16)	0.22
NS	190	0.658		
S	269	0.602		
Sex (F/M ratio)			2.06 (1.27–3.33)	**0.0031**
NS	232 (184/48)	3.81		
S	293 (260/33)	7.62		

*LAC, lupus anticoagulant. Odds ratios were calculated for S vs. NS. p-values of statistical significance are in bold fonts*.

The mean plasma protein levels of MBL between the SLE and non-SLE groups were *not* significantly different [S: 0.151 (0.130–0.172) mg/dL, NS: 0.135 (0.116–0.154) mg/dL; *p* = 0.29]. The mean plasma protein level of factor H was slightly lower in the SLE group [49.9 (48.3–51.5) mg/dL] than that in the non-SLE group [52.9 (50.9–54.9) mg/dL; *p* = 0.02].

The mean ACLA-IgG level was significantly *lower* in the SLE than non-SLE [S: 28.2 (22.7–33.7) GPL, NS: 48.8 (38.7–59.0); *p* = 0.0002]. By contrast, the mean ACLA-IgM levels and the frequency for the presence of LAC were similar between the SLE and the non-SLE groups.

Among the aPL subjects, SLE patients had a higher female to male ratio (7.62 to 1) than non-SLE patients (3.81 to 1; *p* = 0.0031).

### Differential Plasma Protein Levels of Complement and ACLA in aPL-Positive Subjects With Thrombosis, SLE, Both Thrombosis and SLE, and Neither Thrombosis Nor SLE

**Figure 1:**
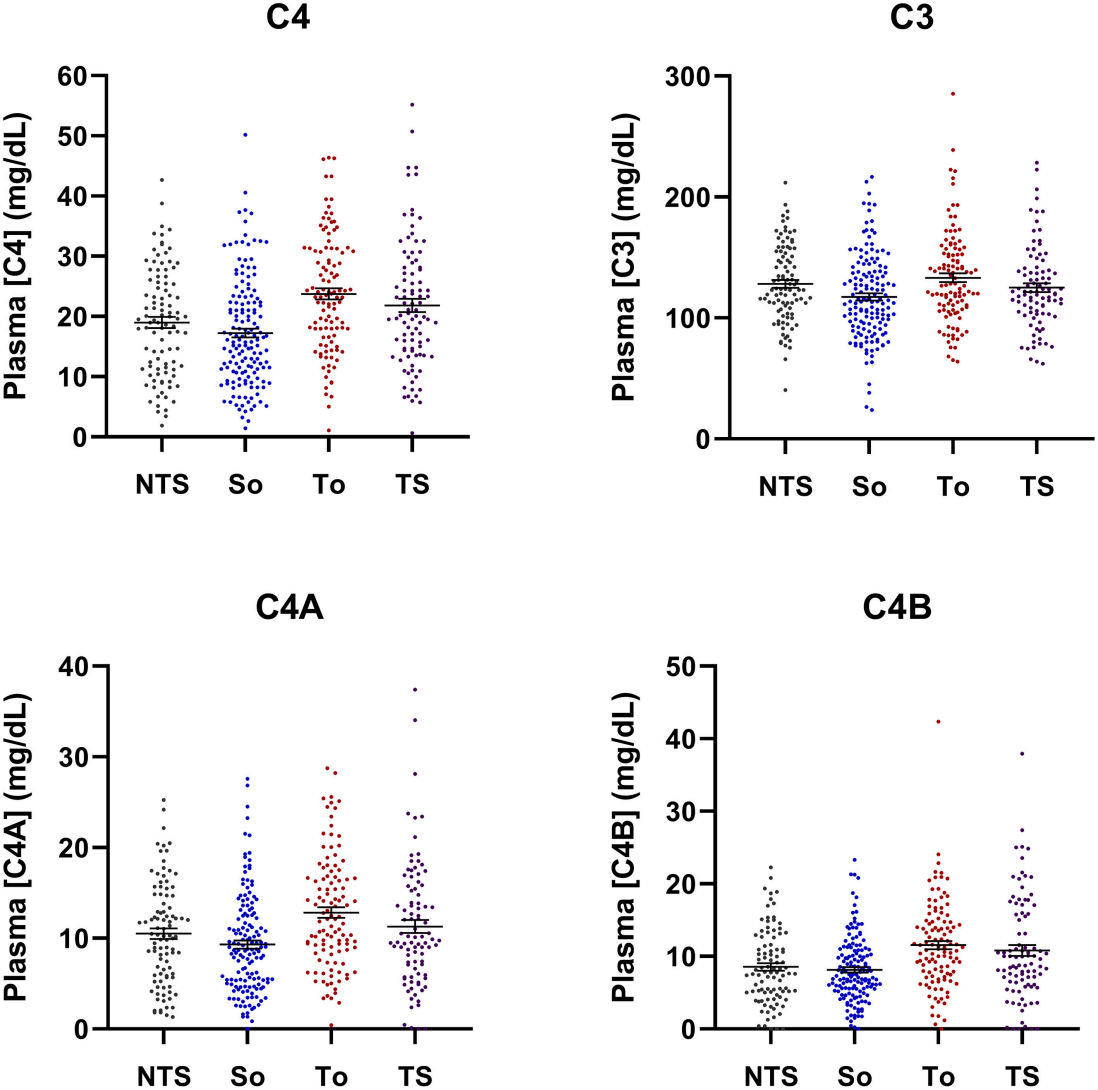
Scattered-plots of complement C4 and C3 plasma protein concentrations in aPL-positive subjects segregated by thrombosis and SLE status and compared. NTS, no thrombosis and no SLE; S_o_, SLE only, T_o_, thrombosis only; TS, with both thrombosis and SLE. Horizontal bars represent means and standard errors. Overall *p*-values are: 2.8 × 10^−7^ for C4; 0.0024 for C3; 5.9 × 10^−5^ for C4A; and 2.7 × 10^−6^ for C4B.

Our study results revealed *higher* levels of mean plasma complement C4, C3, and ACLA-IgG in aPL-positive subjects with a history of thrombosis, but *lower* levels of complement C4, C3, and ACLA-IgG among aPL-positive subjects with SLE. A proportion (37.9%) of aPL-positive subjects diagnosed with SLE also experienced thromboses. To distinguish the roles of complement proteins and ACLA in thromboses and SLE, we segregated the study subjects according to their thrombosis and SLE status: thrombosis only (T_o_), thrombotic SLE (TS), SLE only (S_o_), and no thrombosis and no SLE (NTS) (Figure 1, Table 5). Among these four groups, significantly higher mean protein levels were observed for patients with thrombosis only than with SLE only for plasma protein levels of total C4 (*p* = 2.2 × 10^−9^), C4A (*p* = 1.9 × 10^−6^), C4B (*p* = 1.3 × 10^−6^), C3, (*p* = 2.6 × 10^−5^), ACLA-IgG (*p* = 1.2 × 10^−5^), and in female to male ratio sex ratio (*p* = 1.6 × 10^−6^).

**Table 5:** Mean plasma protein levels of complement and ACLA in aPL-positive subjects segregated by thrombosis and SLE status.

	***n***	**Concentrations**	**95% CI**	**P T_*******o***_: TS**	**T_**o**_: S_*******o***_**	**T_*******o***_: NTS**	**TS: S_*******o***_**	**TS: NTS**	**S_*******o***_: NTS**
C3 protein (RMSE: 35.4)		mg/dL		**0.011**	**2.6** **×** **10**^**−5**^	0.18	0.19	0.26	**0.011**
1. T_o_	126	133.6 ± 37.8	127.0–140.3						
2. TS	110	121.8 ± 34.4	115.3–128.3						
3. S_o_	180	116.1 ± 36.2	110.8–121.5						
4. NTS	104	127.3 ± 32.2	121.0–133.6						
C4 protein (RMSE: 9.72)		mg/dL		0.073	**2.2** **×** **10**^**−9**^	**0.0004**	**0.0001**	0.083	0.056
1. T_o_	126	23.7 ± 9.7	22.0–25.4						
2. TS	109	21.4 ± 11.5	19.2–23.6						
3. S_o_	178	16.8 ± 9.0	15.5–18.1						
4. NTS	104	19.1 ± 8.8	17.4–20.8						
C4A protein (RMSE: 6.12)		mg/dL		0.247	**1.9** **×** **10**^**−6**^	**0.017**	**0.0009**	0.23	**0.049**
1. T_o_	126	12.5 ± 5.9	11.5–13.5						
2. TS	107	11.6 ± 7.6	10.1–13.0						
3. S_o_	171	9.0 ± 5.6	8.2–9.9						
4. NTS	102	10.5 ± 5.6	9.5–11.6						
C4B protein (RMSE: 5.46)		mg/dL		**0.045**	**1.3** **×** **10**^**−6**^	**0.0008**	**0.012**	0.19	0.31
1. T_o_	126	11.2 ± 5.3	10.3–12.2						
2. TS	108	9.8 ± 6.7	8.5–11.0						
3. S_o_	179	8.1 ± 4.9	7.4–8.8						
4. NTS	104	8.8 ± 5.2	7.8–9.8						
									
CFH protein (RMSE:13.7)		mg/dL		0.059	0.78	0.121	0.079	**0.001**	0.053
1. T_o_	111	51.5 ± 13.6	49.0–54.1						
2. TS	96	47.9 ± 9.7	46.0–49.9						
3. S_o_	157	51.1 ± 14.6	48.8–53.4						
4. NTS	92	54.5 ± 15.4	51.3–57.7						
MBL protein (RMSE: 0.154)		mg/dL		0.95	**0.0041**	0.078	**0.0046**	0.076	0.42
1. T_o_	114	0.118 ± 0.140	0.092–0.144						
2. TS	100	0.117 ± 0.115	0.094–0.139						
3. S_o_	152	0.173 ± 0.192	0.143–0.204						
4. NTS	90	0.157 ± 0.137	0.128–0.185						
ACLA-IgM protein (RMSE: 35.7)		g/L		0.28	**0.0039**	**0.013**	0.092	0.092	0.95
1. T_o_	101	16.8 ± 24.7	11.9–21.7						
2. TS	101	22.3 ± 39.9	14.4–30.1						
3. S_o_	172	29.8 ± 39.5	23.9–35.8						
4. NTS	82	30.1 ± 33.4	22.8–37.5						
ACLA-IgG protein (RMSE: 56.9)		g/L		**0.0063**	**1.2** **×** **10**^**−5**^	0.058	0.17	0.488	**0.041**
1. T_o_	103	56.0 ± 87.7	38.9–73.1						
2. TS	103	34.3 ± 38.5	26.7–41.8						
3. S_o_	170	24.5 ± 49.9	17.0–32.0						
4. NTS	84	40.1 ± 37.8	31.9–48.3						
LAC-positivity (Frequency)				0.65	**0.0002**	**0.015**	**3.8** **×** **10**^**−5**^	**0.0048**	0.39
1. T_o_	102	0.735							
2. TS	97	0.763							
3. S_o_	172	0.512							
4. NTS	88	0.568							
Sex (F/M ratio)				**0.0005**	**1.6** **×** **10**^**−6**^	**3.4** **×** **10**^**−7**^	0.43	0.088	0.26
1. T_o_	126	2.07							
2. TS	111	6.40							
3. S_o_	182	8.58							
4. NTS	106	14.1							

*p-values of statistical significance are in bold fonts*.

For functional MBL and the presence of LAC, main differences were observed between the thrombotic groups and the non-thrombotic groups. Mean MBL protein levels were significantly lower in T_o_ and TS than in S_o_ (T_o_ vs. S_o_, *p* = 0.0041; TS vs. S_o_, *p* = 0.0046). The mean level of MBL in aPL-positive subjects without thrombosis and SLE (NTS) was slightly higher than those of T_o_ and TS (*p* = 0.078 and 0.076), and similar to that of S_o_ (*p* = 0.416). For lupus anticoagulant, 73.5% of patients in T_o_ and 76.3% of patients in TS were tested positive, compared to 51.2% in S_o_ (T_o_ vs. S_o_, *p* = 0.0002; TS vs. S_o_, *p* = 0.000038) and 56.8% in NTS (T_o_ vs. NTS, *p* = 0.015; TS vs. NTS, *p* = 0.0048).

### Gene Copy Number Variations of Total *C4, C4A*, and *C4B* in Patients With aPL

Gene copy numbers (GCN) for total *C4, C4A*, and *C4B* from 472 aPL-positive subjects were elucidated by TaqMan based real-time PCR using genomic DNA (35). The copy number of total *C4* genes in this study cohort varied from 2 to 6; *C4A* from 0 to 5; and *C4B* from 0 to 4. The distribution of total *C4, C4A*, and *C4B* gene copy number variations among T_o_, TS, S_o_, and NTS are shown in Table 6.

Table 6Gene copy number variations (CNVs) of total *C4, C4A* and *C4B* among aPL-positive subjects.

**Table:** 

	**T**_****o****_ **(*****N*** **=** **109)**		**TS (*****N*** **=** **100)**		**S**_****o****_ **(*****N*** **=** **166)**		**NTS (*****N*** **=** **98)**		***p***
**GCN**	***N***	***f***	***N***	***f***	***N***	***f***	***N***	***f***	
**A**. C4 CNVs of aPL-positive subjects segregated by thrombosis and SLE status.									
***Total C4***									0.088
2	1	0.009	6	0.060	5	0.030	2	0.020	
3	29	0.266	35	0.350	51	0.307	29	0.296	
4	72	0.661	57	0.570	96	0.578	57	0.582	
5	7	0.064	2	0.020	14	0.084	8	0.082	
6	0	0	0	0	0	0	2	0.020	
***C4A***									**0.034**
0	0	0	4	0.040	1	0.006	1	0.010	
1	23	0.211	23	0.230	36	0.217	16	0.163	
2	72	0.661	61	0.610	106	0.639	57	0.582	
3	14	0.128	12	0.120	23	0.139	19	0.194	
4	0	0	0	0	0	0	4	0.041	
5	0	0	0	0	0	0	1	0.010	
***C4B***									0.13
0	1	0.009	1	0.010	4	0.024	5	0.051	
1	22	0.202	27	0.270	35	0.211	30	0.306	
2	77	0.706	69	0.690	116	0.699	57	0.582	
3	9	0.083	3	0.030	11	0.066	5	0.051	
4	0	0	0	0	0	0	1	0.010

**Table:** 

**B**. Mean gene copy numbers (±SD) for total C4, C4A and C4B among aPL-positive subjects.				
	***N***	**total** ***C4*** **GCN**	***C4A*** **GCN**	***C4B*** **GCN**
***a. Thrombosis (T) and non-thrombosis (NT)***				
NT	263	3.741 ± 0.378	1.989 ± 0.696	1.753 ± 0.633
T	209	3.670 ± 0.613	1.866 ± 0.636	1.804 ± 0.541
	*P*	0.24	**0.049**	0.36
***b. SLE (S) and non-SLE (NS)***				
NS	206	3.782 ± 0.637	2.014 ± 0.702	1.767 ± 0.636
S	266	3.654 ± 0.656	1.872 ± 0.643	1.782 ± 0.561
	p	**0.035**	**0.022**	0.79
***c. Thrombosis and SLE status***				
1. T_o_	109	3.780 ± 0.567	1.917 ± 0.579	1.862 ± 0.552
2. TS	100	3.550 ± 0.642	1.810 ± 0.692	1.740 ± 0.524
3. S_o_	166	3.717 ± 0.659	1.910 ± 0.611	1.807 ± 0.582
4. NTS	97	3.784 ± 0.710	2.124 ± 0.807	1.660 ± 0.705
T_o_ vs. TS	*P*	**0.011**	0.25	0.14
T_o_ vs. S_o_	*P*	0.43	0.93	0.45
T_o_ vs. NTS	*P*	0.97	**0.027**	**0.015**
TS vs. S_o_	*P*	**0.042**	0.24	0.37
TS vs. NTS	*P*	**0.012**	**0.001**	0.34
S_o_ vs. NTS	*P*	0.42	**0.012**	0.052

*T_o_, thrombosis without SLE; TS, thrombotic SLE; S_o_, SLE without thrombosis; NTS, non-thrombosis and non-SLE; f, frequency. GCN, gene copy number. The reference values for mean GCNs of total C4, C4A, and C4B are 3.82 ± 0.75, 2.09 ± 0.79, and 1.74 ± 0.63, respectively, for healthy subjects; and 3.56 ± 0.77, 1.81± 0.89, and 1.76 ± 0.58, respectively, for SLE subjects (38). p values < 0.05 were in bold fonts*.

The distribution of GCN groups was analyzed first as categorical data. The distribution of *C4A* genes was statistically different among T_o_, TS, S_o_, and NTS (*p* = 0.034, χ^2^ analysis). Variations of GCNs for total *C4* (*p* = 0.088) and *C4B* (*p* = 0.13) had not reached statistical significance. The median GCN groups for total *C4* is 4, and for *C4A* and *C4B* are both 2. Low and high copy number groups are defined as those below and above median GCN groups, respectively. Variations in frequencies were observed for the low and high GCN groups of *C4* genes. For example, 41.0% of the TS group had 2 or 3 copies of total *C4* genes (low GCN), compared to 27.5% in T_o_ and 31.6% in NTS. By contrast, only 2.0% of the TS group had 5 or 6 copies of total *C4* (high GCN), compared to 10.2% in the NTS group. A similar pattern was observed for *C4A* genes. There was an increase in the frequency of low *C4A* GCN (27.0% in TS, 17.3% in NTS), and a decrease in the frequency of high *C4A* GCN in the TS group (12.0% in TS, 24.5% in NTS).

The GCN values were analyzed as continuous data to compare means by Student's *t*-test. The means for *total C4, C4A*, and *C4B* were 3.67, 1.87, and 1.80, respectively, for the thrombotic subjects; and were 3.74, 1.99, and 1.75, respectively, for the non-thrombotic subjects. Lower mean *C4A* gene copy number was observed in the thrombotic group (*p* = 0.049) (Table 6).

The mean GCN for total *C4, C4A, and C4B* were 3.65, 1.87, and 1.78, respectively, for the SLE patients; and were 3.78, 2.01, and 1.77, respectively, for non-SLE subjects. Significantly lower mean GCNs for total *C4* and *C4A* were present in the SLE group (*p* = 0.035 for total *C4*; *p* = 0.022 for *C4A*) (Table 6B). The mean *C4B* gene copy numbers were almost identical between patients with and without SLE.

When the aPL subjects were segregated and compared based on both thrombosis and SLE status, it revealed that the NTS group without thrombosis and SLE had the highest mean GCNs for total *C4* and *C4A* (3.78 and 2.12, respectively), but the lowest *C4B* mean GCN (1.66). The thrombotic SLE group (TS) had the lowest values of total *C4* at 3.55 and *C4A* at 1.81.

The mean GCN of total *C4* for NTS was significantly higher than that of TS (*p* = 0.012)*;* of *C4A* for NTS was significantly higher than those of T_o_ (*p* = 0.027), S_o_ (*p* = 0.012) and TS (*p* = 0.001); and of *C4B* was significantly lower than that of T_o_ (*p* = 0.015).

For TS, the mean GCN of total *C4* was significantly lower than those of T_o_ (*p* = 0.011), S_o_ (*p* = 0.042), and NTS (*p* = 0.012); and of *C4A* was significantly lower than that of NTS (*p* = 0.001).

### Plasma C4 Protein Concentrations Per *C4* Gene Copy in Thrombosis and SLE

**Figure 2:**
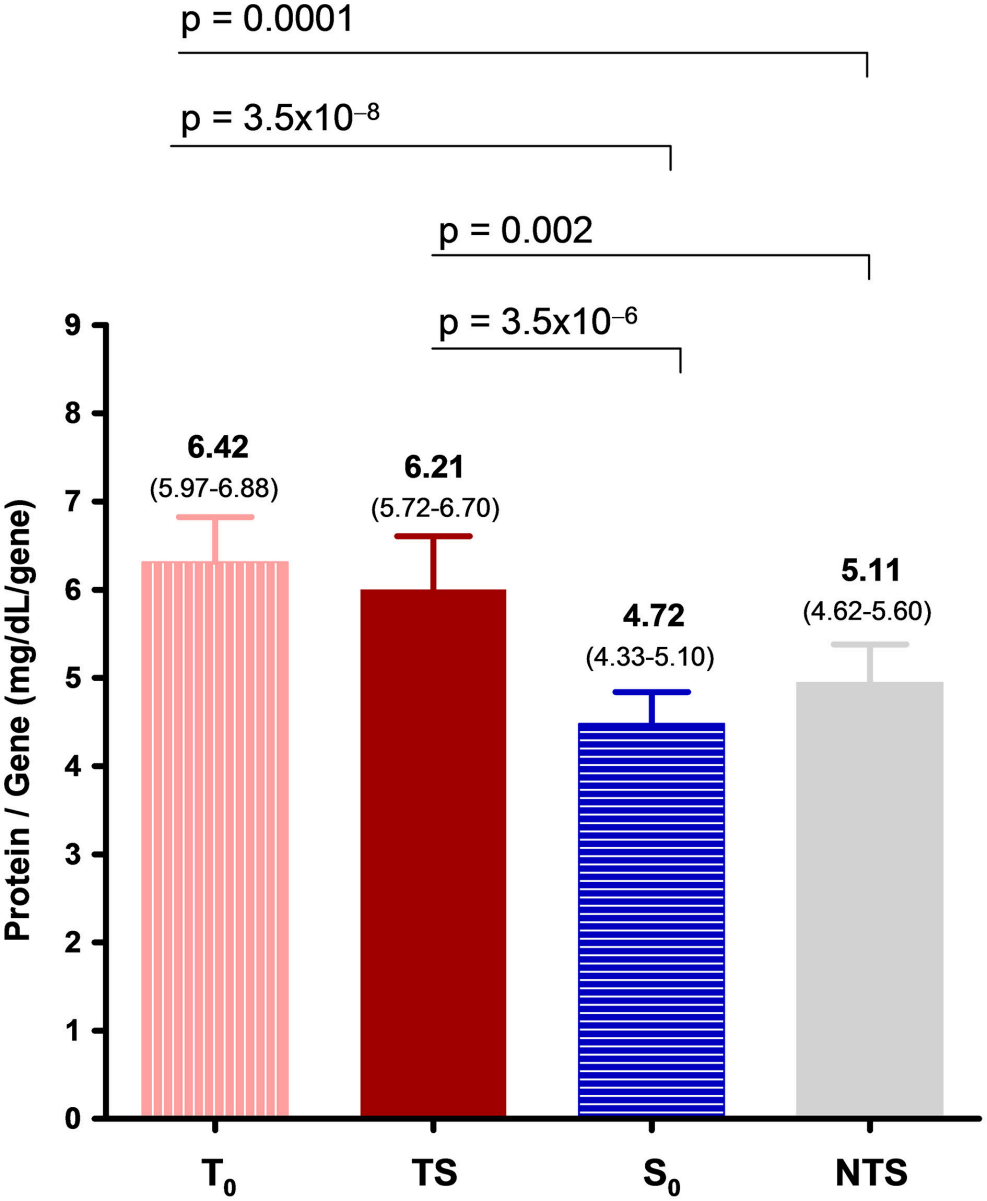
A comparison of mean total plasma C4 protein concentrations per gene-copy (with 95% confidence levels) among aPL-positive subjects categorized by thrombosis and SLE status. Protein concentration per gene-copy allows a comparison of C4 protein levels under various clinical conditions by eliminating the effects of *C4* gene copy number variations among patients.

Both *C4* gene copy number variation and clinical conditions of aPL subjects are important determining factors for C4 plasma protein concentrations. To examine the respective roles of genetic variants and clinical status on plasma protein levels of complement C4, we calculated the C4 protein per gene copy in each study subject by dividing the C4 plasma protein concentration with the *C4* gene copy number. The mean C4 protein concentrations per gene dose among T_o_, TS, S_o_ and NTS were 6.42, 6.21, 4.72, and 5.11 mg/dL, respectively. Thus, the C4 plasma protein yield per gene copy was the highest in aPL subjects with thrombosis only, and the lowest in aPL subjects with SLE only. Highly significant differences were present between the two thrombotic groups (T_o_ and TS) and the two non-thrombotic groups (S_o_ and NTS) (Figure 2).

### Differential Complement and ACLA Plasma Protein Levels and C4 Genetic Deficiencies in aPL-Positive Subjects With and Without Recurrent Pregnancy Loss (RPL)

Of the 444 female aPL-positive subjects, 106 experienced recurrent pregnancy loss (RPL). Thromboses occurred in 63.8% of the RPL patients, compared to 33.7% in non-RPL patients (*p* = 5.1 × 10^−8^). SLE were diagnosed in 54.3% of the RPL patients and 59.8% in non-RPL patients (*p* = 0.32). Strikingly, RPL had the highest frequency in patients with both thrombosis and SLE (TS, 41.0%) but lowest in patients with SLE only (S_o_, 13.3%); patients of the NTS and T_o_ groups each had a frequency of 22.9%. The frequencies of RPL are significantly different among aPL-positive patients when segregated into T_o_, S_o_, TS, and NTS (Table 6; χ^2^ = 46.2, degree of freedom = 3, *p* = 3.8 × 10^−10^). TS patients had an odds ratio (95% confidence interval) of 8.63 (4.37–17.0) over S_o_ patients to experience recurrent pregnancy loss (*p* = 1.6 × 10^−11^).

Lupus anticoagulant was present in 67.1% of patients with RPL and 58.4% of non-RPL female patients (*p* = 0.057). Mean total C4 and ACLA-IgG protein levels were significantly increased, while mean CFH level was reduced (Table 7) among RPL patients. The mean total C4 level was 21.8 (19.9 ± 23.7) mg/dL in RPL and 19.1 (18.1 ± 20.2) mg/dL in non-RPL (*p* = 0.015). The ACLA-IgG was 44.2 (35.1 ± 53.3) g/l in RPL and 30.0 (24.2 ± 35.8) g/l in non-RPL (*p* = 0.01). The CFH mean concentrations in RPL were 48.8 (46.6 ± 51.0) mg/dL and 52.1 (50.5 ± 53.7) mg/dL in non-RPL (*p* = 0.019).

**Table 7:** Plasma complement and ACLA levels and risk factors for recurrent pregnancy loss (RPL) in *female* aPL-positive subjects.

***a. Continuous data***		***n***	**Mean ± SD**	**95% CI**	***P***
C3 Protein, mg/dL	N-RPL	336	123.6 ± 34.0	120.0–127.3	0.23
	RPL	105	131.8 ± 41.8	123.1–140.4	
C4 protein, mg/dL	N-RPL	334	19.2 ± 9.9	18.1–20.2	**0.009**
	RPL	103	22.1 ± 9.6	19.9–23.7	
C4A protein	N-RPL	324	10.3 ± 5.9	9.6–10.91	**0.039**
	RPL	103	11.7 ± 5.5	10.5–12.8	
C4B protein	N-RPL	333	9.1± 5.5	8.5–9.7	0.051
	RPL	104	10.3 ± 5.8	9.2–11.4	
CFH protein, mg/dL	N-RPL	296	52.1 ± 14.2	50.5–53.7	**0.019**
	RPL	87	48.8 ± 10.4	46.6–51.0	
MBL protein, mg/dL	N-RPL	269	0.166 ± 0.164	0.147–0.185	0.051
	RPL	84	0.128 ± 0.130	0.100–0.156	
ACLA-IgM g/L	N-RPL	300	26.8 ± 37.2	22.6–31.0	0.56
	RPL	90	33.6 ± 108.5	10.9–56.4	
ACLA IgG, g/L	N-RPL	301	30.0 ± 51.1	24.2–35.8	**0.01**
	RPL	91	44.2 ± 43.7	35.1–53.3	
***b. Categorical data***		***Case/total***	***f***	***P***	**Remarks**
*C4T*, GCN = 2	N-RPL	7/261	0.0269	0.15	
	RPL	5/79	0.0633		
*C4A*, GCN = 0	N-RPL	0/263	0	**0.0001**	Risk
	RPL	5/81	0.0617		
*C4B*, GCN = 0	N-RPL	11/261	0.0421	**0.017**	Protective
	RPL	0/75	0		
LAC-Positivity	N-RPL	168/294	0.584	0.057	
	RPL	64/94	0.671		
Thromboses	N-RPL	114/338	0.337	**5.1** **×** **10**^**−8**^	Risk
	RPL	67/105	0.638		
SLE	N-RPL	136/338	0.414	0.32	
	RPL	48/105	0.467		
**c. Subgroup freq**.	**T**_***o***_	**S**_***o***_	**TS**	**NTS**	***p***
N-RPL, *n* (%)	61 (18.1)	149 (44.1)	53 (15.7)	75 (22.2)	**3.8** **×** **10**^**−10**^
RPL, *n* (%)	24 (22.9)	14 (13.3)	43 (40.1)	24 (22.9)	
S_o_, *p*	**7.2** **×** **10**^**−5**^	-			
TS, *p*	**0.021**	**1.6** **×** **10**^**−11**^	-		
NTS, *p*	0.54	**0.0006**	**0.0024**	-	

*f, frequency; N-RPL, no recurrent pregnancy loss; RPL, with recurrent pregnancy loss. p-values of statistical significance are in bold fonts*.

Homozygous *C4A* deficiency (GCN of *C4A* = 0) was present in five female aPL patients and all of these five subjects experienced RPL (*p* = 0.0001). On the contrary, homozygous *C4B* deficiency (GCN of *C4B* = 0) was present in 11 female aPL patients and *none* of them experienced RPL (*p* = 0.017). Thus, homozygous *C4A* deficiency was a strong risk factor for, and homozygous *C4B* deficiency was a strong protective factor against, recurrent pregnancy loss.

### Standardized Comparison of Numeric Parameters Associated With Thrombosis and SLE

**Figure 3:**
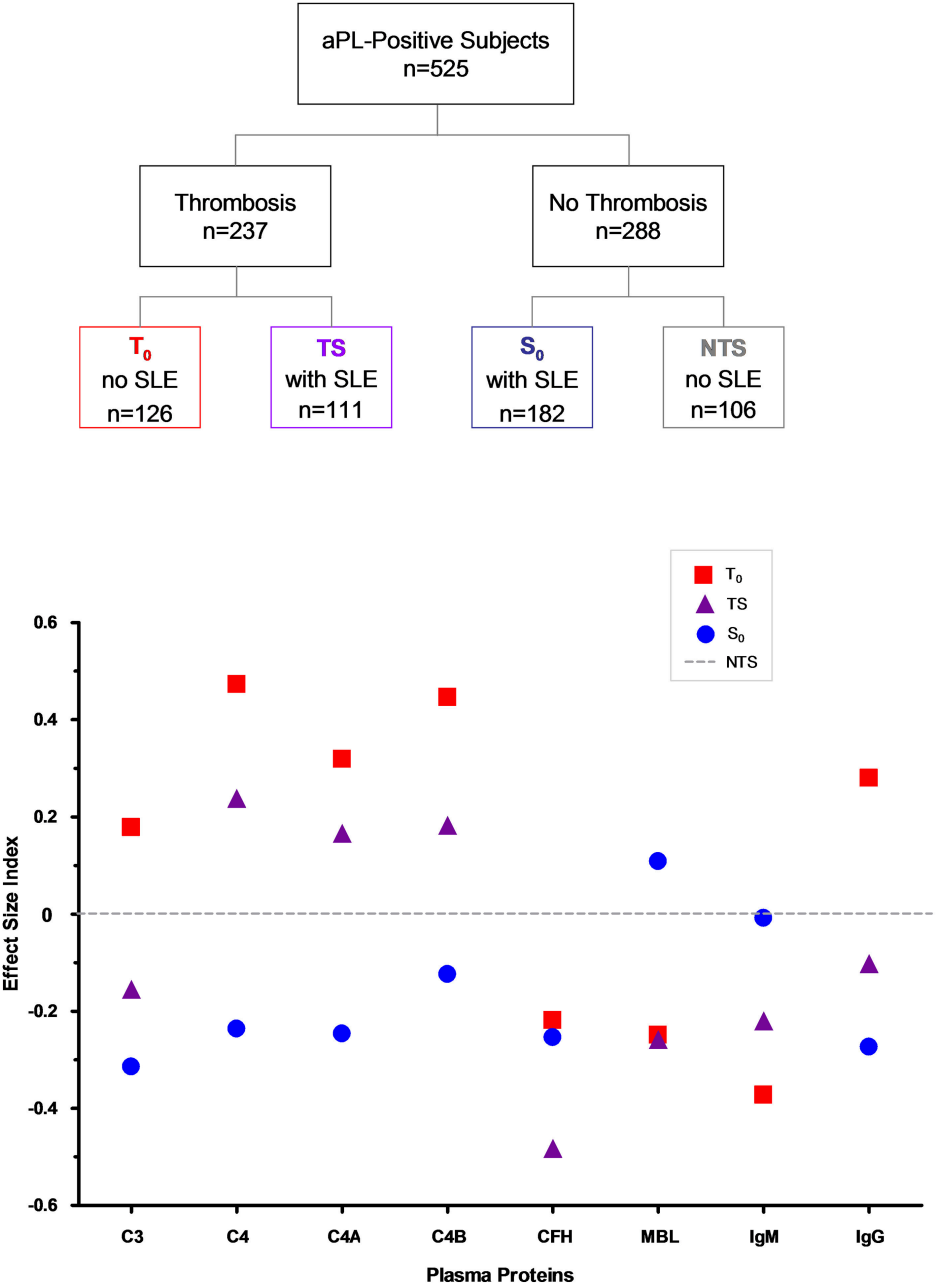
**(Upper)** Categorization of aPL-positive subjects according to their thrombosis and SLE status. **(Lower)** Standardized differences (effect size index) of mean complement protein levels and ACLA levels among aPL-positive subjects with thrombosis without SLE (T_o_), thrombosis and SLE (TS), SLE without thrombosis (S_o_) when compared to the non-thrombotic and non-SLE (NTS) group. Root mean square error (RMSE) value for each protein was derived from Oneway ANOVA. Differences of mean protein levels for T_o_, TS and S_o_ from NTS were each divided by their associated RMSE and charted to derive the effect size index. The effect size indices allow a standardized comparison of different parameters under different clinical conditions.

To allow a standardized comparison of complement and ACLA protein variations in thrombosis and SLE, we determined the effect size index (69) of each parameter in T_o_, S_o_, and TS, using the mean protein concentrations or antibody levels of the group without thrombosis and SLE (NTS) as a reference. The difference of mean protein levels for each protein at T_o_, S_o_, or TS from NTS was divided by its root mean square error (RMSE), which was computed by ANOVA, to yield the effect size index. The effect size indices for complement and ACLA proteins with quantitative variations are depicted in Figure 3. In descending order, the greatest intergroup effect size indices are: C4, T_o_ vs. S_o_: 0.709; C4B, T_o_ vs. S_o_: 0.570; C4A, T_o_ vs. S_o_: 0.566; ACLA-IgG, T_o_ vs. S_o_: 0.554; complement factor H, TS vs. NTS: 0.483; ACLA-IgM, T_o_ vs. NTS: 0.373; and MBL, TS vs. S_o_: 0.367.

## Discussion

This is a cross-sectional study of complement protein profiles and copy number variations of *C4* genes in a relatively large cohort of human subjects with aPL antibodies. These aPL-positive subjects had a variety of clinical presentations: thrombosis, SLE, thrombosis and SLE, no thrombosis and no SLE. Many female aPL-positive subjects also experienced pregnancy morbidity such as recurrent pregnancy losses. Extensive analyses of gene copy number variations for total *C4, C4A*, and *C4B*, plasma protein levels of total C4, C4A, C4B, C3, factor H and MBL, and antiphospholipid antibodies revealed distinct patterns of diversity that can be relevant and effective quantitative biomarkers for thrombosis, SLE and recurrent pregnancy loss.

An intriguing aspect of complement *C4* genetics is the *frequent* gene copy number variations (30, 38, 40). Genetic deficiency (60, 70) or low gene copy numbers of total *C4* or *C4A* has been shown to be a prevalent risk factor for SLE in European and East-Asian Americans (22, 38, 41, 71–73). *C4* gene copy number variations in the aPL-positive subjects were determined and validated by quantitative real-time PCR (35). When SLE and non-SLE subjects were compared, lower total *C4* and *C4A* mean gene copy numbers were found among the SLE subjects, suggesting that aPL-positive subjects with low total *C4* or *C4A* gene copy numbers carried a greater risk of developing SLE, as we reported in earlier studies (22, 38). Among the NTS subjects who were not afflicted with thrombosis and SLE, there were higher mean GCN of *C4A*, which would be protective against SLE; and low GCN of *C4B* that would lead to lower C4B protein levels and thereby reducing the risk of thrombosis.

Using thrombosis as a response, multiple logistic regression analyses suggested that *higher* plasma C4 protein levels and the presence of lupus anticoagulant (LAC) were among the strongest independent biomarkers associated with thrombosis (C4, *p* = 6.2 × 10^−9^; LAC, *p* = 6.9 × 10^−5^) (Supplementary Table). Other relevant parameters for increased risk of thrombosis included male sex and a *reduction* of complement factor H level, which was also observed by Nakamura and colleagues (74). Higher plasma C4 protein levels and the presence of LAC were the two most prominent risk factors for *arterial* thrombosis. The higher level of C4 protein in arterial thrombosis was mainly attributable to higher C4B (*p* = 2.1 × 10^−5^). The risk factors identified for *venous* thrombosis also included increased protein level of total C4 (*p* = 0.01) and the presence of LAC (*p* = 0.012). Reduced protein level of functional MBL (*p* = 0.0012) and male gender also had prominent effects.

While the presence of LAC and elevated ACLA-IgG levels have long been recognized for their connections with thrombosis and recurrent pregnancy loss (75), this report provides a firm documentation on the significance of *higher* C4 plasma protein levels among aPL-positive subjects with APS-related clinical manifestations. The presence of LAC and elevated protein level of complement C4 together are predictors for increased risk of thrombosis with values of sensitivity at 0.707 and specificity at 0.664. This study also reveals lower complement factor H protein levels among subjects with SLE and thrombosis. Deficiency, mutation or autoantibody of complement factor H have been linked to atypical hemolytic uremic syndrome that is characterized by thrombotic microangiopathy (76, 77). Along with observations that plasma protein levels of MBL were decreased, evidence for an involvement of complement proteins in human thrombosis or pregnancy loss are compelling and deserve clinical attention (78). High levels of plasma C4 among patients with thrombosis could result in a procoagulation or thromboinflammatory state, which provide large quantities of reagents to fuel the complement cascades, leading to greater extent of complement-mediated tissue injuries. The abundance of the fast-reacting C4B could aggravate the pathogenic process in arterial thrombosis.

Using SLE as a response, multiple logistic regression analysis of plasma protein data suggested that *reduced* levels of C3, C4, and ACLA-IgG, and female gender were strong risk factors for SLE C3 is downstream of C4 in the classical and the MBL activation pathways, the activation and consumption of C3 are amplified by a positive feedback mechanism (79–81). In other words, moderate activation of C4 can lead to large consumption of C3. Thus, fluctuations of serum C3 levels tend to be a more sensitive biomarker for SLE disease activity than C4 does.

Among the female aPL-positive subjects, patients with thrombosis and particularly, thrombotic SLE, had high frequencies of recurrent pregnancy loss. RPL patients had elevated levels of complement C4 and ACLA-IgG, and decreased concentration of factor H. Remarkably, female aPL-positive subjects with homozygous C4B deficiency were *all protected* from RPL, which is consistent with observations in mouse models that complement C4 deficiency or C3 deficiency were protective from RPL induced by injection of human aPL (16). It is also of interest to note that aPL-positive female (human) patients with homozygous C4A deficiency *all* experienced RPL, which underlies the importance of C4A protein in achieving tolerance or defense against autoimmunity and fetal rejections.

It is important to recognize that the direction of changes for plasma protein levels of complement C3, C4, and ACLA-IgG among aPL-positive patients with SLE and with thrombosis or pregnancy morbidity are mostly *opposite* to each other. The highest mean protein levels for these proteins were present in the T_o_ group (thrombosis without SLE), and the lowest in the S_o_ group (SLE without thrombosis). Thus, the inter-group differences of these proteins were highly significant (T_o_ vs. S_o_: *p* = 2.6 × 10^−5^ for C3; *p* = 2.2 × 10^−9^ for C4; *p* = 1.2 × 10^−5^ for ACLA-IgG). The resultant effects for these two opposing forces, SLE and thrombosis, are shown in the thrombotic SLE group TS, by which the mean plasma protein levels of C3, C4, C4A, C4B, and ACLA-IgG all fell between those of T_o_ and S_o_ groups, and their values were closer to those present in the NTS group. When *standardized* by the gene copy numbers, highly significant differences for mean C4 protein concentrations per gene-copy were observed between the thrombotic subjects and non-thrombotic subjects, and the greatest difference remained between T_o_ and S_o_ (6.42 mg/dL/gene for T_o_, 4.72 mg/dL/gene for S_o_; *p* = 3.5 × 10^−8^). The mean C4 protein per gene-copy in TS (6.21 mg/dL/gene) was only slightly lower than that of T_o_. This implies the presence of trans-acting factor(s) among patients with thrombosis that *upregulates* C4 protein biosynthesis, and/or reduces its turnover that would have decreased the protein levels. While complement activation is a noted feature for clinical manifestations of APS, such activation likely occurs *locally* that may *not* result in systemic and parallel decline of plasma protein levels for C4 and C3, a phenomenon analogous to what we observed in many patients with juvenile dermatomyositis (82).

The target sites for most aPL appear to be located at the domain D1 or complement controlling protein repeat on β_2_GPI. Recombinant antibody recognizing this domain D1 induced fetal loss and coagulation in animal models (83). Interestingly, an engineered β_2_GPI antibody without the IgG heavy chain CH_2_-domain, which was devoid of the C1q binding site and unable to fix or activate complement, was shown to compete and control the coagulation and abortive effects in animals burgeoned by injection of human aPL (83). Biochemical studies revealed that β_2_GPI in its linear conformation can serve as a regulator for the classical and alternative pathway C3 convertases, as it diminished the activation of C3 (to form C3a) and the assembly of C5b-9 in a dose-dependent manner. Active β_2_GPI also enhanced the degradation of C3b in the presence of factor I and factor H (84, 85). The effects of aPL on the functional activities of β_2_GPI and plasma complement protein concentrations and activities remain to be elucidated.

The relationships among MBL deficiency, SLE and thrombosis were complex and it was not clear whether MBL deficiency was a risk factor for SLE (86, 87). In a study of 91 SLE patients, Garred et al. demonstrated a near doubling of thrombosis in individuals homozygous for MBL protein structural variants (B,D,C) that led to functional deficiencies of MBL (88). Subsequently, an association was made between MBL deficiency and arterial thrombosis (89). In a study of structural variants and promoter alleles for high and low expression of *MBL2* gene in 114 SLE patients, Font et al. observed that low MBL expression genotypes were associated with venous thrombosis (90). Data from our study further clarifies the role of MBL in SLE and thrombosis: reduced plasma protein concentrations of *functional* MBL were present among aPL-positive patients with thrombosis, regardless of SLE status. Therefore, the link between MBL-deficiency (or low expression of MBL) and SLE could be secondary to *low* functional MBL in SLE patients with APS. As a lectin binding protein that binds to simple carbohydrate (mannose) components on cell membranes, it is possible that MBL could compete with aPL for binding to phospholipids or phospholipid-binding proteins to reduce the risk of aPL on initiating thrombotic events.

Our study population includes multiple racial and ethnic backgrounds but the majority were of Northern European ancestry. When we analyzed the complement and ACLA data on this specific ethnic group, similar conclusions on the contrasting patterns of complement C4 and ACLA in thromboses and SLE can be reached. Results on three clinical studies on stroke or recurrent pregnancy loss were in accord with our observations that *high* C4 and/or *high* C3 plasma protein levels are associated with thrombosis or recurrent pregnancy loss (91–93).

Our observations are consistent with a parallel and independent study that revealed that pediatric SLE patients undergoing a clinical trial (94) with a history of hypertension had persistently higher serum levels of complement C4 and C3 and higher gene copy number of *C4B* (Mulvihill et al, submitted). Here, we further show that patients with both SLE and thrombosis had the lowest mean GCNs for total *C4* (3.55) and *C4A* (1.81), which underscores the importance of C4A deficiency as a genetic risk factor for systemic autoimmune disease. Paradoxically, hypocomplementemia is both a cause and an effect of human SLE. SLE-associated disorders such as lupus nephritis, hemolytic anemia, high titers of anti-dsDNA, and lupus disease flares are notably marked by low serum complement levels due to massive consumption of C3 and C4 (21–23, 25). Systemic and concurrent consumptions of C4 and C3 can be reflected by higher coefficients of correlation (*r* or *r*^2^) between these two proteins, which are conspicuous among aPL-positive patients in the S_o_ and NTS groups (Table 2) (25). Detailed diagnostic disorders of SLE (64) and triple positivity of aPL autoantibodies (9) were not available for this study to examine the extent of hypocomplementemia in various organ involvement and tissue damage, but these would be relevant topics for future investigations.

This cross-sectional study represents a snapshot of complement and aPL in a population of human subjects with antiphospholipid antibodies. The relatively large study population provided an informative dataset to examine specific patterns of complement and aPL among patients with thrombosis, SLE and recurrent pregnancy loss. Along time courses of patients with chronic, systemic autoimmune disease, plasma or serum complement C3 and C4 levels and their cell-bound products would fluctuate with disease activities. The status of SLE/APS disease activities including flare and remission for each patient at the time of sample collection was not available and therefore not accounted for in our data analyses. The lack of data from longitudinal studies, and blood samples from healthy subjects with and without aPL being processed in parallel with similar methodologies are other limitations of this study. Further studies with large sample size of patients for measurements of complement component protein levels under defined genetic backgrounds, plus determination of activation products C3a, C4a, and C5a, cell-bound and fluid phase levels of C4d and C3d, and membrane attack complexes may provide important insights into mechanism(s) on how complement modulate aPL associated clinical manifestations and disease activities of SLE and APS (55, 58, 61, 62). In addition, effects of complement-mediated tissue damage and thromboses would be more readily demonstrated by immunohistochemical methods.

In conclusion, our results can serve as a foundation for further studies of SLE and APS disease mechanisms, more sensitive disease diagnosis, and possibly better prognosis of disease course and profile. It would be desirable to elucidate the *C4* gene copy numbers among aPL-positive subjects for a prevention purpose, as those with low total *C4* or *C4A* gene copy number would have a higher risk to develop SLE, and high *C4B* GCN would have greater risk for complement-mediated complications such as thrombosis, recurrent pregnancy loss in females, and tissue injuries.

## Author Contributions

C-YY, SS, YW, and RR designed the research. RR contributed patient samples and clinical data. SS, KK, DZ, YW, and C-YY performed experiments. HN, YW, SS, and C-YY performed statistical analyses. SS, YW, DZ, EM, FB-S, SA, RR, and C-YY analyzed and interpreted data. SS, YW, KK, DZ, EM, FB-S, SA, RR, HN, and C-YY wrote the paper.

### Conflict of Interest Statement

The authors declare that the research was conducted in the absence of any commercial or financial relationships that could be construed as a potential conflict of interest.

## Abbreviations

ANOVA: analysis of variance
aPL: antiphospholipid antibodies
APS: antiphospholipid syndrome
CNV: copy number variation
GCN: gene copy number
LAC: lupus anticoagulant
MBL: mannan binding lectin
NS: no SLE
NT: no thrombosis
NTS: no thrombosis and no SLE
RMSE: root mean square error
RPL: recurrent pregnancy loss
S: SLE
S_o_: SLE without thrombosis
SLE: systemic lupus erythematosus
T, thrombosis, T_o_: thrombosis without SLE
TS: thrombosis and SLE.
