# Supplementary material for: Opposite Profiles of Complement in Antiphospholipid Syndrome (APS) and Systemic Lupus Erythematosus (SLE) Among Patients With Antiphospholipid Antibodies (aPL)
Source: Front Immunol. 2019 May 7;10:885. doi: 10.3389/fimmu.2019.00885 (PMC6514053; doi:10.3389/fimmu.2019.00885)
Supplement: Supplementary file 1 [file Table_1.pdf]

Opposite Profiles of Complement in Antiphospholipid Syndrome (APS) and Systemic Lupus Erythematosus (SLE) among Patients with Antiphospholipid Antibodies (aPL)

Stephanie L. Savelli,[1,2,4] Robert A.S. Roubey,[5] Kathryn J. Kitzmiller,[1,4] Danlei Zhou,[1,3,4] Haikady N. Nagaraja,[6] Evan Mulvihill, [3,4] Fatima Barbar-Smiley,[3,4] Stacy P. Ardoin, [3,4] Yee Ling Wu,[1,4,7] and C. Yung Yu [1,3,4]

[1]The Research Institute at Nationwide Children's Hospital, [2]Division of Hematology/Oncology, [3]Division of Rheumatology, Nationwide Children's Hospital, 700 Children's Drive, Columbus Ohio 43205; [4]Department of Pediatrics, College of Medicine, The Ohio State University, Columbus, OH; [5]Division of Rheumatology, Allergy and Immunology, The University of North Carolina at Chapel Hill, Chapel Hill, NC; [6]Division of Biostatistics, College of Public Health, The Ohio State University, Columbus, OH and [7]Department of Microbiology and Immunology, Loyola University Chicago, IL

Supplementary Table

Supplementary Table. Multiple logistic regression analyses of independent parameters related to thromboses, recurrent pregnancy loss and SLE in aPL-positive subjects.

A. Thrombosis

Whole model summary: n=395, DF=5; $\chi^2 = 74.1; p = 1.47 \times 10^{-14}$

**a. Effect Likelihood Ratio Tests**		
Source	χ^2	*p*
C4	33.8	6.2×10^{-9}
LAC	15.8	0.000069
CFH	10.0	0.0016
SLE	6.88	0.0087
Gender	6.46	0.011
**b. Odds Ratios**		
Parameter	*Odds Ratio*	*Remarks*
C4 (mg/dL)	1.07	higher C4, ↑risk
C4 (range)	45.1	
LAC (+/ -)	2.56	LAC +, ↑ risk
CFH () mg/dL	0.97	higher CFH, protective
CFH (range)	0.045	
SLE (+/-)	0.55	SLE +, protective
Gender (F/M)	0.46	female, protective

B. Arterial Thrombosis

Whole model summary: n=452, DF=2; $\chi^2 = 26.6; p = 1.7 \times 10^{-6}$

**a. Effect Likelihood Ratio Tests**		
Source	χ^2	*p*
C4B	18.1	2.2×10^{-5}
LAC	8.33	0.0027
**b. Odds Ratios**		
Parameter	*Odds Ratio*	*Remarks*
C4B (mg/dL)	1.09	higher C4, ↑risk
C4B (range)	11.0	
LAC (+/ -)	2.23	LAC +, ↑ risk

C. Venous Thrombosis

Whole model summary: n=395, DF=4; $\chi^2 = 36.8; p = 2.0 \times 10^{-7}$

**a. Effect Likelihood Ratio Tests**		
Source	χ^2	*p*
MBL	10.5	0.0012
Gender (M)	8.42	0.0037
C4	6.62	0.010
LAC	6.33	0.012

**b. Odds Ratios**		
Parameter	*Odds Ratio*	*Remarks*
MBL(mg/dL)	0.997	higher MBL, protective
MBL (range)	0.059	
LAC (+/ -)	1.84	LAC +, ↑ risk
C4 (mg/dL)	1.03	higher C4, ↑risk
C4 (range)	4.86	
Gender (M)	2.31	male, ↑risk

**D. Recurrent pregnancy loss**		
Whole model summary: n=336, DF=3; χ^2 = 14.0; p=0.0029		
**a. Effect Likelihood Ratio Tests**		
Source	χ^2	*p*
C4	6.69	0.0097
ACLA-IgG	4.76	0.029
CFH	4.59	0.032
**b. Odds Ratios**		
Term	*Odds Ratio*	*Remarks*
C4 (mg/dL)	1.04	higher C4, ↑ risk
C4 (range)	7.21	
ACLA IgG (GPL)	1.01	higher ACLA-IgG, ↑ risk
ACLA IgG (range)	32.8	
CFH (mg/dL)	0.976	higher CFH, protective
CFH (range)	0.070	

**E. Nominal Logistic Fit for SLE (with C3)**		
Whole model summary: n=457, DF=3; χ^2 = 40.81; p=7.2 x 10^{-9}		
**a. Effect Likelihood Ratio Tests**		
Source	χ^2	*p*
C3	19.2	1.2 x 10^{-5}
ACLA IgG	17.3	3.2 x 10^{-5}
Gender	6.03	0.014
**b. Odds Ratios**		
Term	*Odds Ratio*	*Remarks*
C3 (mg/dL)	0.988	low C3, ↑ risk
C3 (range)	0.042	
ACLA IgG (GPL)	0.991	low ACLA-IgG, ↑ risk
ACLA IgG (range)	0.0033	
Gender (F/M)	1.98	female, ↑ risk

**F. Nominal Logistic Fit for SLE (with C4)**		
Whole model summary: n=456, DF=3; $\chi^2 = 33.47$*; p=2.6 x* 10^{-7}		
**a. Effect Likelihood Ratio Tests**		
Source	χ^2	*p*
ACLA IgG	15.0	0.0001
C4	12.7	0.0004
Gender	4.82	0.028
**b. Odds Ratios**		
Term	*Odds Ratio*	*Remarks*
ACLA IgG (GPL)	0.992	low ACLA-IgG, ↑ risk
ACLA-IgG (range)	0.0058	
C4 (mg/dL)	0.966	low C4, ↑ risk
C4 (range)	0.147	
Gender (F/M)	1.84	female, ↑ risk
